# The comprehensive immunomodulation of NeurimmiRs in haemocytes of oyster *Crassostrea gigas* after acetylcholine and norepinephrine stimulation

**DOI:** 10.1186/s12864-015-2150-8

**Published:** 2015-11-14

**Authors:** Hao Chen, Lingling Wang, Zhi Zhou, Zhanhui Hou, Zhaoqun Liu, Weilin Wang, Dahai Gao, Qiang Gao, Mengqiang Wang, Linsheng Song

**Affiliations:** Key Laboratory of Experimental Marine Biology, Institute of Oceanology, Chinese Academy of Sciences, Qingdao, China; Key Laboratory of Mariculture & Stock enhancement in North China’s Sea, Ministry of Agriculture, Dalian Ocean University, Dalian, 116023 China; University of Chinese Academy of Sciences, Beijing, China

**Keywords:** Oyster, NeurimmiR, Acetylcholine, Norepinephrine, Immunomodulation, Neural-endocrine-immune system

## Abstract

**Background:**

Neural-endocrine-immune (NEI) system is a major modulation network among the nervous, endocrine and immune system and weights greatly in maintaining homeostasis of organisms during stress and infection. Some microRNAs are found interacting with NEI system (designated NeurimmiRs), addressing swift modulations on immune system. The oyster *Crassostrea gigas*, as an intertidal bivalve, has evolved a primary NEI system. However, the knowledge about NeurimmiRs in oysters remains largely unknown.

**Results:**

Six small RNA libraries from haemocytes of oysters stimulated with acetylcholine (ACh) and norepinephrine (NE) were sequenced to identify neurotransmitter-responsive miRNAs and survey their immunomodulation roles. A total of 331 miRNAs (132 identified in the present study plus 199 identified previously) were subjected to expression analysis, and twenty-one and sixteen of them were found ACh- or NE-responsive, respectively (FDR < 0.05). Meanwhile, 21 miRNAs exhibited different expression pattern after ACh or NE stimulation. Consequently, 355 genes were predicted as putative targets of these neurotransmitter-responsive miRNAs in oyster. Through gene onthology analysis, multiple genes involved in death, immune system process and response to stimulus were annotated to be modulated by NeurimmiRs. Besides, a significant decrease in haemocyte phagocytosis and late-apoptosis or necrosis rate was observed after ACh and NE stimulation (*p* < 0.05) while early-apoptosis rate remained unchanged.

**Conclusions:**

A comprehensive immune-related network involving PRRs, intracellular receptors, signaling transducers and immune effectors was proposed to be modulated by ACh- and NE-responsive NeurimmiRs, which would be indispensable for oyster haemocytes to respond against stress and infection. Characterization of the NeurimmiRs would be an essential step to understand the NEI system of invertebrate and the adaptation mechanism of oyster.

**Electronic supplementary material:**

The online version of this article (doi:10.1186/s12864-015-2150-8) contains supplementary material, which is available to authorized users.

## Background

Neural-endocrine-immune (NEI) system, defined firstly by Besedovsky and Sorkin in 1977, is a closely interacted network among the nervous, endocrine and immune system [1]. These three systems communicate actively with each other by releasing neurotransmitters, hormones and cytokines, respectively [2]. The well balanced NEI system and reciprocal regulation inside are essential for organisms to maintain homeostasis during the stress or infection [2]. Acetylcholine (ACh) and norepinephrine (NE) have been considered as representative neurotransmitters in parasympathetic and sympathetic nervous system, respectively [3, 4], and they contribute an intensive modulation on the immune response of mammals during stress and bacteria challenge [5, 6].

Recently, microRNAs (miRNAs) have been identified as an important mechanism to modulate the NEI system in mammals. As a class of endogenously encoded single-strand non-coding RNAs, miRNAs have been suggested as vital regulators in gene expression by binding to their 3’-UTR [7]. To date, a great number of miRNAs have been identified from diverse species, with striking conservation in their secondary structure and length (~22 nt) [8]. It has been estimated that miRNAs could be involved in the modulation of almost all biological processes, including growth, development, cell differentiation and immunity [7, 9].

Accumulating evidences has recently demonstrated that some miRNAs (designated NeurimmiRs) could act as “negotiators” within the NEI system and function indispensably in maintaining the immune homeostasis during infection [10–12]. For example, miR-132, a well-known NeurimmiR in mammals, could repress the expression of acetylcholinesterase after LPS stimulation, resulting in the attenuation of the vagus nerve and the release of tumor necrosis factor and interleukin-6 [11]. Other eight miRNAs were also identified as NeurimmiRs, which shared close connection with various neuropathologies [10]. However, less is known about NeurimmiRs in invertebrates who might own more preliminary NEI system than vertebrates.

The Mollusca is a large diverse phylum in invertebrates and possess the ancient neuroendocrine system, such as ancient cholinergic and catecholaminergic system, similar to that in mammals [13, 14]. The ancient molluscan neuroendocrine system, can be activated by bacteria stimulation and modulate the immune response in turn [15]. As an intertidal bivalve, oysters *Crassostrea gigas* are continuously suffered by environmental stress, which attracts more attention to the balance of NEI system. The available genome information [16] and the knowledge of immune mechanism [17] as well as some previously identified miRNAs [18] make it suitable to investigate the modulation of NEI system in oyster [19]. The purposes of the present study were to (1) further identify neurotransmitter-induced NeurimmiRs from oyster, (2) characterize miRNA expression patterns after the neurotransmitter stimulation, (3) survey the potential modulation of NeurimmiRs on the immune response of oyster, hopefully providing new hints for the dynamic regulation within the NEI system in mollusc.

## Result

### Overview of small RNA sequencing

Six small RNA libraries constructed from corresponding samples in PBS control group, ACh stimulation group and NE stimulation group were sequenced by Ion Torrent Proton. Totally, 50.0 M, 57.4 M, 54.7 M, 60.6 M, 67.9 M and 63.6 M raw reads were obtained from those six libraries, respectively (Additional file 1: Table S1). After discarding the disqualified reads and incorporating identical reads, 1.9 M, 2.0 M, 1.6 M, 2.1 M, 2.3 M and 2.5 M unique reads were obtained correspondingly (Additional file 1: Table S1). Two peaks were observed in the length distribution (Additional file 2: Figure S1) of all the unique reads (8.6 M) obtained from these six libraries. A total of 715,372 reads remained with copy number more than 6, and were applied for the subsequent alignment with Rfam database and oyster mRNA. Finally, 519,571 reads were retained for further mapping to the genome (Additional file 3: Table S2).

### The miRNAs identified from oyster haemocytes

**Fig. 1:**
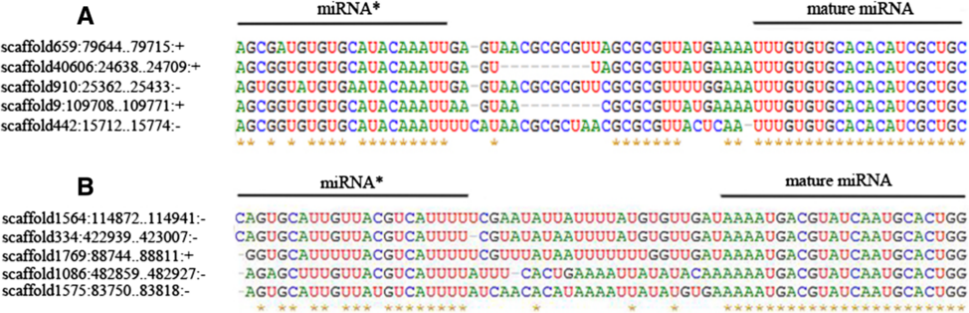
Precursor sequence alignment of miRNAs having five potential coding regions. **a** scaffold659_26519; **b** scaffold 1564_13662. The left panel represents genomic location of the pri-miRNA including the scaffold number, position of the first and last nucleotide, and direction (“+” indicates sense strand, “-” indicates antisense strand), respectively

To identify miRNAs expressed in oyster haemocytes after neurotransmitter stimulation, a total of 6,820 reads which have at least one alignment in genome, were subjected to miRDeep2 software for prediction of the precursor sequence and secondary structure. A total of 132 new miRNAs including 5 known ones and 127 novel ones were identified (Additional file 4: Table S3), among which, 27 miRNAs possessed at least two precursor-coding regions (Fig. 1, Additional file 5: Table S4). Considering 199 miRNAs (Additional file 4: Table S3) identified previously, a total of 331 miRNAs were discovered in oyster haemocytes. Within those miRNAs, 76 miRNAs shared the same seed sequence with miRNAs identified in other species (designated known miRNAs) and 255 ones possessed new seed sequence (designated novel miRNAs).

### Differentially expressed miRNAs after neurotransmitter stimulation

**Fig. 2:**
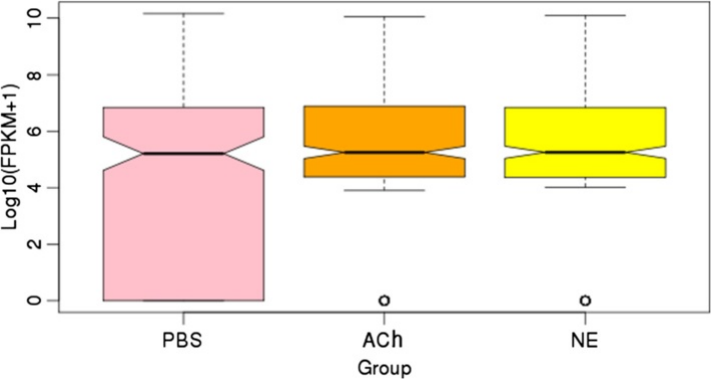
Expression level of all miRNAs in three groups. Pink: control group; Orange: ACh stimulation group; Yellow: NE stimulation group. The bottom and top of the box represents the first and third quartiles of log_10_(FPKM + 1) values in corresponding group while the line insides the box stands for the median value. The overall expression level of oyster miRNAs in ACh and NE stimulation groups were significantly higher than that in PBS control group

Copy numbers of the 331 miRNAs identified in oyster haemocytes were counted and further converted to fragments per kilo base of transcript per million fragments mapped (FPKM) value to analyze their expression level (Additional file 6: Table S5). FPKM distributions of total miRNAs in those three groups were analyzed and depicted in box plot (Fig. 2). The bottom and top of the box represented the first and third quartiles of log_10_(FPKM + 1) values in corresponding group while the line insides the box stood for the median value. Though the median and third quartile in PBS, ACh and NE groups were similar, the first quartile of control group was significantly lower than that in neurotransmitter stimulation groups (Fig. 2).

**Fig. 3:**
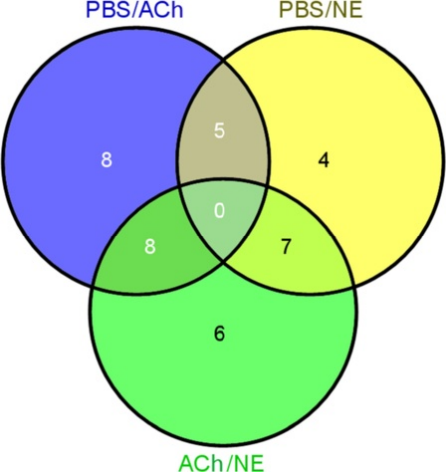
Grouping of differentially expressed miRNAs. Blue circle represents miRNAs expressed differentially in ACh group when compared with PBS group. Yellow circle indicates differentially expressed miRNAs in PBS/NE comparison while green indicates that in ACh/NE comparison

Differentially expressed miRNAs after neurotransmitter stimulation were determined subsequently by edgeR software with FDR less than 0.05 (Additional file 7: Table S6). As a result, a sum of 21 miRNAs were found expressed differentially in the ACh stimulation group compared to that in the PBS control group, including 18 increased and three decreased ones (Fig. 3). Ten miRNAs were up-regulated after NE stimulation while six were down-regulated, compared with that in PBS group (Fig. 3). Five miRNAs (cgi-miR-125, cgi-miR-8, scaffold1144_2255, scaffold1711_599 and scaffold226_18954) were found responsive to both the ACh and NE stimulation. Furthermore, 21 miRNAs showed different expression pattern in ACh and NE stimulation groups, among which, eight miRNAs exhibited higher expression level in NE stimulation group and 13 miRNAs exhibited higher expression level in ACh stimulation group (Fig. 3).

**Fig. 4:**
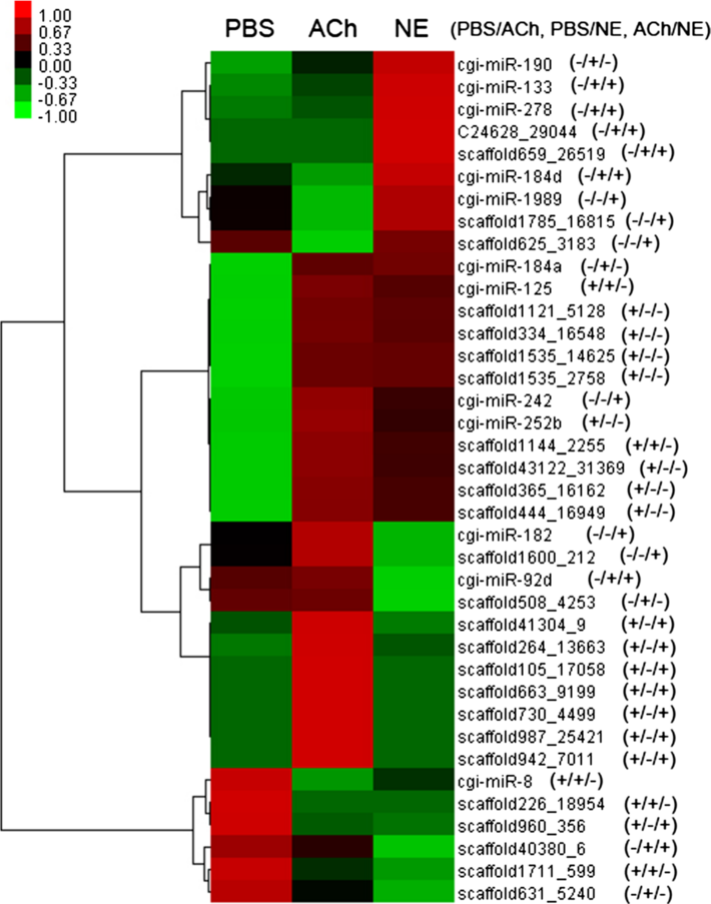
Heat map and hierarchical clustering of differentially expressed miRNAs. Neurotransmitter-responsive miRNAs were clustered with converted FPKM value by Cluster 3.0 in default settings. Converted expressions of certain miRNA were shown with different colors. The “+” or “-” symbol after each miRNA ID indicates its significance state of expression difference in PBS/ACh, PBS/NE, ACh/NE comparison with “+” represents significant difference and “-” represents no significant difference

Those 38 neurotransmitter-responsive miRNAs were then clustered by Cluster3.0 using FPKM value (Fig. 4). Among them, four miRNAs (cgi-miR-184d, cgi-miR-1989, scaffold785_16815 and scaffold625_3183) decreased merely in ACh group and seven miRNAs (scaffold41304_9, scaffold264_13663, scaffold105_17058, scaffold663_9199, scaffold730_4499, scaffold942_7011 and scaffold987_25421) increased strictly after ACh stimulation. Meanwhile, nine miRNAs responded exclusively to NE stimulation including five (cgi-miR-190, cgi-miR-133, ci-miR-278, C24628_29044 and scaffold659_26519) up-regulated and four (cgi-miR-182, cgi-miR-92d, scaffold1600_212, and scaffold508_4253) down-regulated.

### Gene onthology (GO) terms of target genes of neurotransmitter-responsive miRNAs

**Fig. 5:**
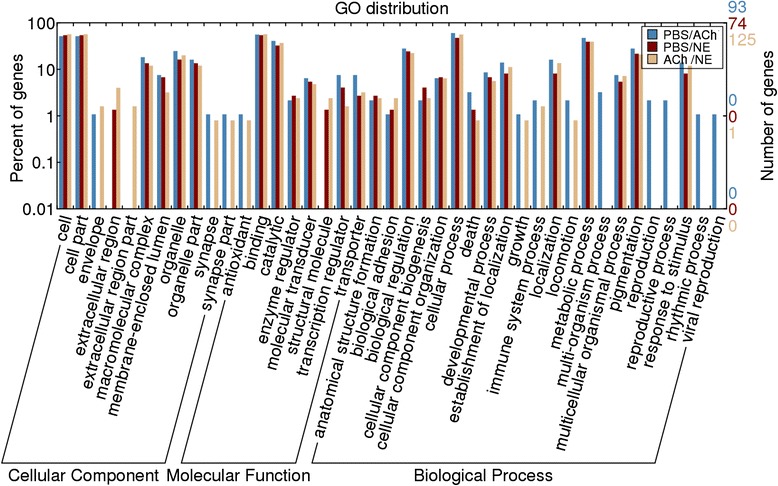
GO distribution of target genes of differentially expressed miRNAs computed by WEGO. Blue: target genes of differentially expressed miRNAs between the control and ACh stimulation group; Dark red: differentially expressed miRNAs between the control and NE stimulation group; Orange: differentially expressed miRNAs between the ACh and NE stimulation groups

A total of 355 oyster genes were predicted as putative targets of those neurotransmitter-responsive miRNAs by miRanda software (Additional file 8: Table S7). The GO term distributions of all target genes were analyzed by Blast2GO software and displayed through Web Gene Ontology Annotation Plot (WEGO) (Fig. 5). Finally, immune-related biological process (death, immune system process and response to stimulus) and molecular function (antioxidant) were annotated from those targets of neurotransmitter-responsive miRNAs.

### Immune-related genes targeted putatively by NeurimmiRs

**Fig. 6:**
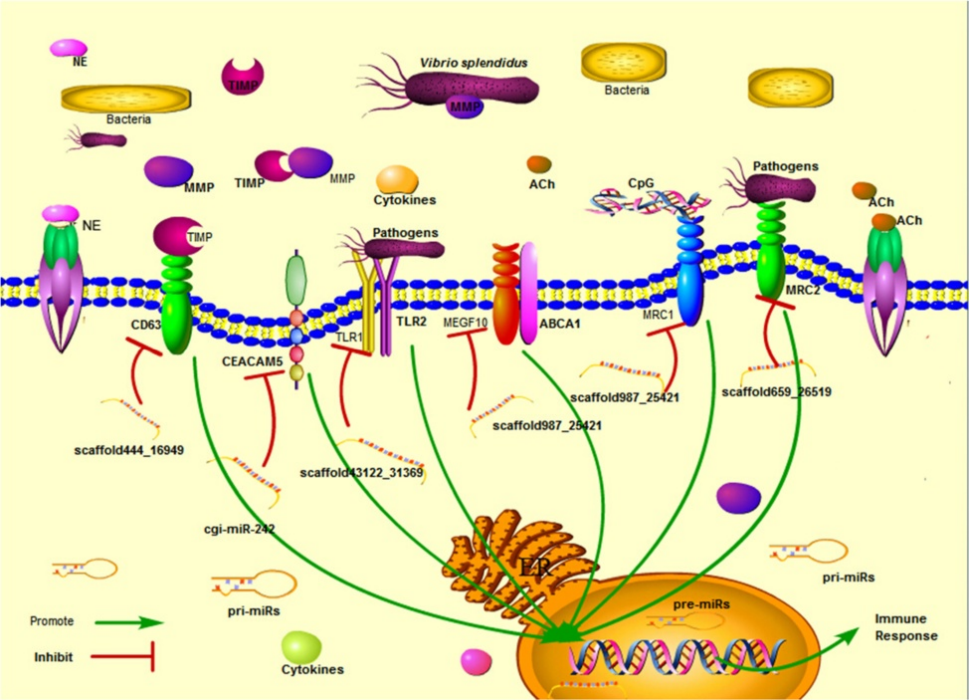
Diverse receptors were predicted to be modulated by NeurimmiRs. PRRs and intracellular receptors such as MRC1, MRC2, TLR1, CEACAM5, MEGF10 and CD63 were targeted by diverse NeurimmiRs. Supportive molecules such as ACh, ACh receptor, NE, NE receptor, TIMP, MMP (metalloproteinase), TLR2 (Toll like receptor 2) and ABCA1 (ABC transporter A family member 1) and cellular component such as cell membrane, nucleus and endoplasmic reticulum (ER) were illustrated for better understanding

**Fig. 7:**
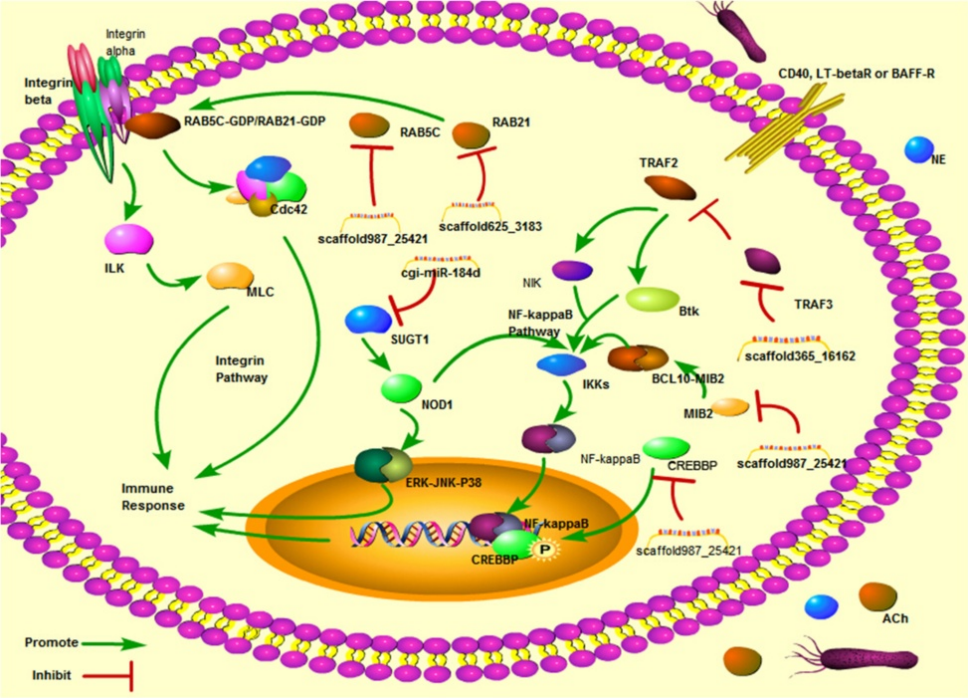
NeurimmiRs were found targeting multiple molecules of the integrin and NF-κB pathway. Diverse signaling molecules, such as RAB-5C, RAB-21, SUGT1, TRAF3, MIB2, CREBBP in integrin and NF-κB pathway were predicted to be targets of NeurimmiRs. Molecules such as ILK (integrin linked kinease), MLC (Myosin light-chain), NIK (NF-KappaB-inducing kinase), IKKs (IkappaB kinases) and cellular component such as cell membrane and nucleus were also demonstrated as reference

**Fig. 8:**
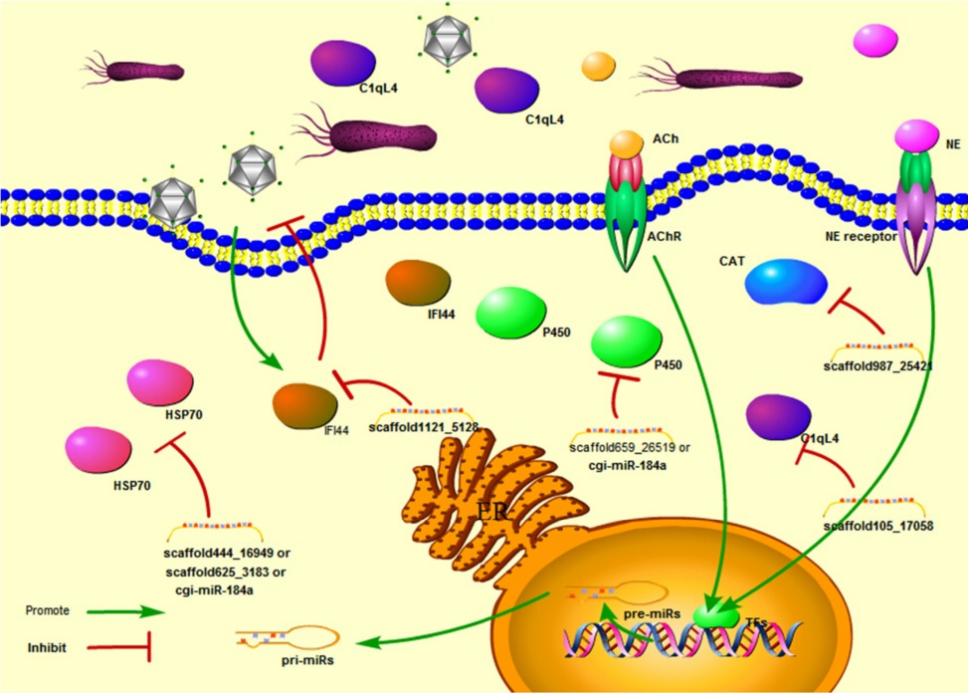
Immune effectors targeted by NeurimmiRs. Immune effectors such as HSP70, P450, IFI44, CAT and C1qL4 were also annotated as putative targets of NeurimmiRs responsive to ACh and NE. Cellular component such as cell membrane, nucleus and ER were illustrated as well

Diverse immune-related genes involved in pathogen recognition, signaling transduction, immune and stress responses were predicted as putative targets for those neurotransmitter-responsive miRNAs (Table 1). For example, the transcripts of pathogen recognition receptor (PRRs), such as mannose receptor 1 (MRC1, CGI_10013598), C-type mannose receptor 2 (MRC2, CGI_10003643), Toll like receptor 1 (TLR1, CGI_10021136), carcinoembryonic antigen-related cell adhesion molecule 5 (CEACAM5, CGI_10010080), and intracellular receptors, such as multiple epidermal growth factor-like domains 10 (MEGF10, CGI_10022484), muscarinic acetylcholine receptor (mAChR, CGI_10026715), nicotinic acetylcholine receptor (nAChR CGI_10012300), CD63 antigen (CD63, CGI_10024556), were prognosticated to be modulated by some NeurimmiRs (Fig. 6). Some signal transducers annotated as CREB-binding protein (CREBBP, CGI_10004570), Ras-related protein Rab-21 (RAB-21, CGI_10012407), Ras-related protein Rab-5C (RAB-5C, CGI_10016941), E3 ubiquitin-protein ligase mib2 (MIB2, CGI_10012437), TNF alpha-induced protein 3 (TNFAIP3, CGI_10016154), and TNF receptor-associated factor 3 (TRAF3, CGI_10019401) were also suggested to be the targets of NeurimmiRs by miRanda (Fig. 7). Immune and stress effectors such as HSP70 (CGI_10006158, CGI_10014234, CGI_10014235 and CGI_10027276), cytochrome P450 (CGI_10012275, CGI_10011491) and interferon-induced protein 44 (IFI44, CGI_10011953) were also suggested to be manipulated by certain NeurimmiRs (Fig. 8).

**Table 1:** Immune-related genes putatively targeted by NeurimmiRs

Classification	Target Gene	Gene ID	NCBI Accession Number	miRNA	miRNA Expression
Receptors	carcinoembryonic antigen-related cell adhesion molecule 5 (CEACAM5)	CGI_10010080	EKC30453	cgi-miR-242	ACh (+^1^)
	C-type mannose receptor 2 (MRC2)	CGI_10013598	EKC36613	scaffold987_25421	ACh (+)
	Toll like receptor 1 (TLR1)	CGI_10021136	EKC32484	scaffold43122_31369	ACh (+)
	multiple epidermal growth factor-like domains 10 (MEGF10)	CGI_10022484	EKC30915	scaffold987_25421	ACh (+)
	CD63 antigen (CD63)	CGI_10024556	EKC30322	scaffold444_16949	ACh (+)
	muscarinic acetylcholine receptor (mAChR)	CGI_10026715	EKC38948	scaffold1535_2758	ACh (+)
	macrophage mannose receptor 1 (MRC1)	CGI_10003643	EKC21607	scaffold659_26519	NE (+)
	nicotinic acetylcholine receptor (nAChR)	CGI_10012300	EKC27791	cgi-miR-1989	NE (+)
Signaling molecules	CREB-binding protein (CREBBP)	CGI_10004570	EKC21118	scaffold987_25421	ACh (+)
	Ras-related protein Rab-21 (RAB-21)	CGI_10012407	EKC30126	scaffold625_3183	ACh (−^2^)
	E3 ubiquitin-protein ligase mib2 (MIB2)	CGI_10012437	EKC24345	scaffold987_25421	ACh (+)
	Ras-related protein Rab-5C (RAB-5C)	CGI_10016941	EKC27651	scaffold987_25421	ACh (+)
	TNF receptor-associated factor 3 (TRAF3)	CGI_10019401	EKC37852	scaffold365_16162	ACh (+)
	TNF alpha-induced protein 3 (TNFAIP3)	CGI_10016154	EKC18030	cgi-miR-1989	NE (+)
	suppressor of G2 allele of SKP1 homolog (SUGT1)	CGI_10016471	EKC33616	cgi-miR-184d	NE (+)
Immune effector	Catalase (CAT)	CGI_10003354	EKC25821	scaffold987_25421	ACh (+)
	heat shock 70 kda (HSP70)	CGI_10006158	-	scaffold625_3183	ACh (−)
	interferon-induced protein 44 (IFI44)	CGI_10011953	EKC23550	scaffold1121_5128	ACh (+)
	HSP70	CGI_10014234	-	scaffold444_16949	ACh (+)
	HSP70	CGI_10014235	XP_011426978	scaffold444_16949	ACh (+)
	complement C1q-like protein 4 (C1qL4)	CGI_10026904	EKC37620	scaffold105_17058	ACh (+)
	cytochrome p450 (P450)	CGI_10011491	EKC33448	scaffold659_26519	NE (+)
	P450	CGI_10012275	EKC18482	cgi-miR-184a	NE (+)
	HSP70	CGI_10027276	EKC43179	cgi-miR-184a	NE (+)

^1^miRNA increased in corresponding group

^2^miRNA decreased in corresponding group

### Changes of phagocytosis and apoptosis rate of oyster haemocytes after neurotransmitter stimulation

**Fig. 9:**
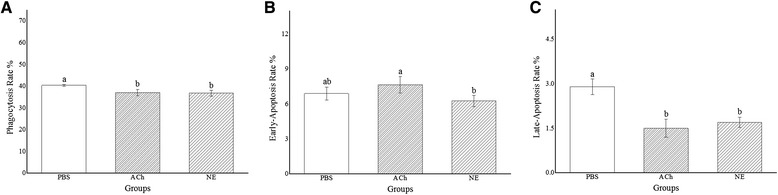
Changes of haemocyte phagocytosis and apoptosis rate by ACh and NE. **a** Phagocytosis rate, **b** early-apoptosis rate and **c** late-apoptosis and necrosis rate of haemocytes from oysters stimulated with ACh and NE were determined using flow cytometry

The phagocytosis and apoptosis rate of oyster haemocytes after neurotransmitter stimulation were evaluated to survey the immunomodulation role of ACh and NE. Both the phagocytosis rate in the haemocytes of ACh and NE group dropped significantly when compared with that in control group (*P* < 0.05) (Fig. 9a). No remarkable changes of early-apoptosis rate was found in the ACh and NE groups compared with control group (Fig. 9b) while the late-apoptosis and necrosis rate decreased significantly (*P* < 0.05) (Fig. 9c).

## Discussion

As a class of gene regulator at post-transcription level, miRNAs are usually transcribed by RNA polymerase II and incorporated into RNA-induced silencing complex where they can binds to the 3’-UTR of target genes and induce their translational repression or degradation [20]. Though diverse miRNAs have been identified in regulating immune genes, fewer reports investigated their interactions with neurotransmitter [10]. In the present study, six miRNA libraries from oyster haemocytes after ACh and NE stimulation were sequenced to identify neurotransmitter-responsive miRNAs and further investigate their potential involvements in the modulation of immune response. A total of 132 new miRNAs were identified herein, including 5 known ones and 127 novel ones. In addition with 199 miRNAs identified previously, a total number of 331 miRNAs have been discovered in oyster *C. gigas* so far. Among those, 255 miRNAs failed to be mapped to any mature miRNAs in the miRBase database, suggesting that they might be oyster-specific. The total number of miRNAs in *C. gigas*, was larger than that in other molluscs such as *Haliotis rufescens* [21], *Lotti agigantea* [21] and *Pinctada martensii* [22], indicating that they might play indispensable roles in the thrive of oysters in intertidal environment.

ACh and NE have been proved to be vital neurotransmitters in the response against stress and infection [2, 3, 23, 24] and the expression of diverse immune-related genes could be fast switched by them (3–12 h) [13, 14], indicating the participation of miRNAs during the modulation. Accordingly, in the present study, 38 of 331 oyster miRNAs depicted intensively expressional alteration at 6 h after ACh and NE stimulation. Among them, two ACh-responsive miRNAs (cgi-miR-125 and cgi-miR-8) and six NE-responsive miRNAs (cgi-miR-125, cgi-miR-133, cgi-miR-190, cgi-miR-278, cgi-miR-8 and cgi-miR-92d) were also found as immune-responsive [18]. Furthermore, some immune-related biological process (death, immune system process and response to stimulus) and molecular function (antioxidant) were annotated from the target genes of those 38 neurotransmitter-responsive miRNAs (Fig. 5), which collectively implied the potential immunomodulation role of the neurotransmitter-responsive miRNAs in oyster. Moreover, those 38 neurotransmitter-responsive miRNAs were clustered into eight distinct branches based on their FPKM value (Fig. 4), where miRNAs in the same branch shared similar expression pattern after the neurotransmitter stimulation. Those miRNAs with similar expression pattern were suspected to function synergistically to maintain the homeostasis of NEI system in oyster. Interestingly, most of the neurotransmitter-responsive miRNAs were newly discovered ones in this species, indicating the existence of novel miRNA regulation mechanism in the NEI system of oyster.

NeurimmiRs represent a class of miRNAs which could modulate the interactions in NEI system [10]. In the present study, multiple genes involved in the pathogen recognition, signaling transduction and immune function were predicted as targets of NeurimmiRs in oyster. PRRs are the first players to initiate the immune response by recognizing pathogens [25]. As invertebrates, oysters have evolved a sophisticated innate immune system with large number of PRRs to survive from harsh environment full of pathogens [16, 17, 26]. It is found in the present study that NeurimmiRs from oyster could modulate some PRRs, such as mannose receptors [27], TLRs [28] and CEACAM5 [29]. Moreover, some intracellular receptors triggering immune response could also be targeted by oyster NeurimmiRs. For example, MEGF10, a *bona fide* homology of engulfment receptor cell death abnormality protein-1 which could promote cellular engulfment by cooperation with ATP binding cassette transporter [30], was annotated as a putative target of scaffold987_25421. Besides, CD63, which could act as the membrane receptor of metalloproteinase inhibitor (TIMP) and specially inhibit metalloproteinase from pathogenic bacteria *Vibrio splendidus* of oyster [31], was also found to be modulated by NeurimmiR scaffold444_16949. Collectively, those results suggest a dynamic regulation of NeurimmiRs in recognizing pathogens and triggering downstream immune signals in oyster.

Meanwhile, multiple signaling transducers and immune effectors were also proposed to be modulated by oyster NeurimmiRs. In the present study, two Rab genes (RAB-21 and RAB-5C), activators in integrin pathway [32], were found to be modulated by NeurimmiR scaffold625_3183 and scaffold987_25421, respectively. Three vital regulators in NF-κB pathway [33, 34] (CREBBP [35], MIB2 [36] and TRAF3 [37]) were also predicated as targets of ACh-responsive NurimmiRs (scaffold987_25421, scaffold987_25421 and scaffold365_16162). However, the expression pattern of scaffold625_3183 and scaffold987_25421 was opposite, while CREBBP, MIB2 and TRAF3 conflicts with each other in activating NF-κB. It was speculated that contradictions in modulating integrin pathway and NF-κB pathway by those NeurimmiRs could render a flexible switch on the immune response of oysters. Moreover, some NurimmiRs could also target the immune effectors in oyster. For example, IFI44, a virus-responsive gene activated by TLR3 to inhibit virus proliferation [38, 39], was annotated as putative target of scaffold1121_5128. Similarly, multiple NE-responsive NeurimmiRs were suggested to manipulate the expression of HSP70 [40], an important regulator in immune response.

**Fig. 10:**
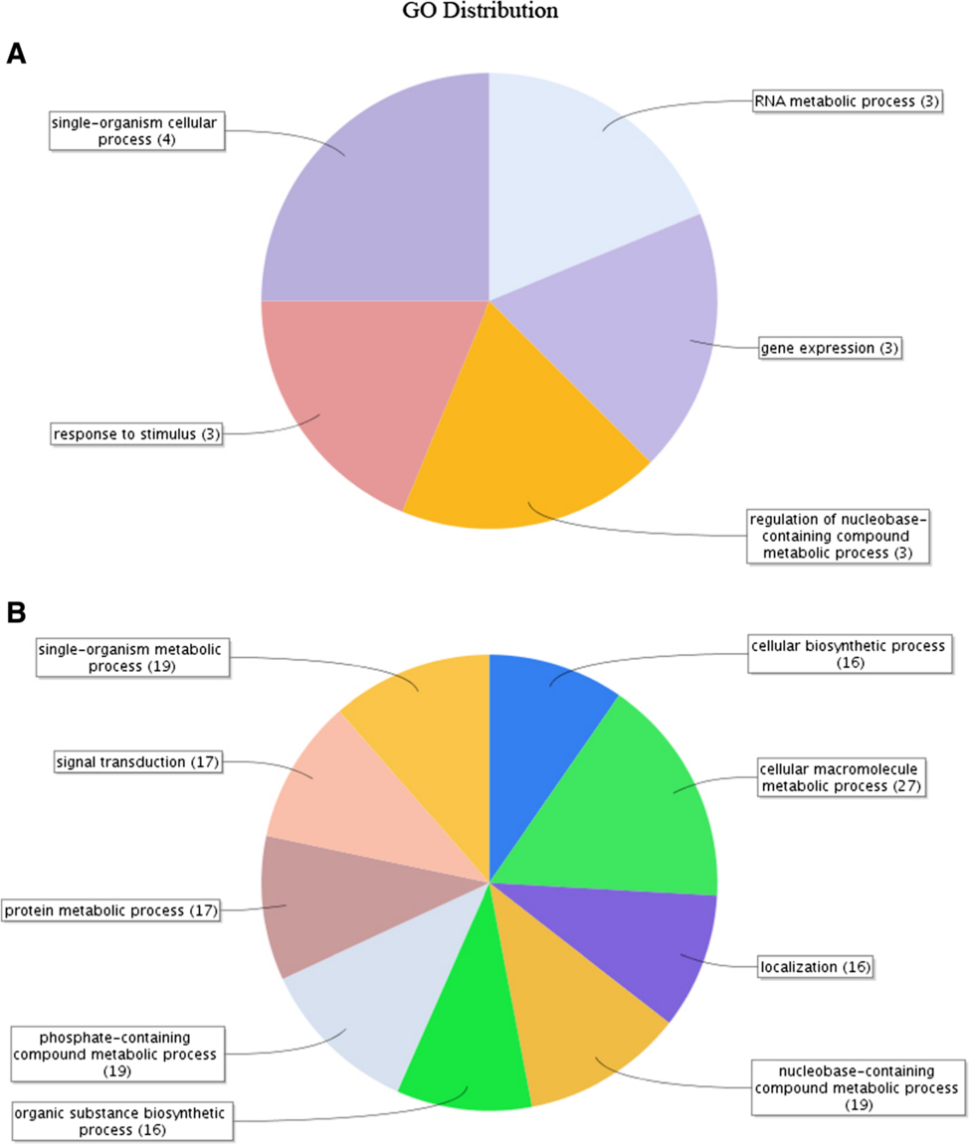
GO distribution of target genes of differentially expressed miRNAs. **a** Target genes of five miRNAs which responded to both ACh and NE stimulation and **b** target genes of 21 miRNAs which were differentially expressed between ACh and NE stimulation group were subjected to Blast2GO for GO distribution analysis

**Fig. 11:**
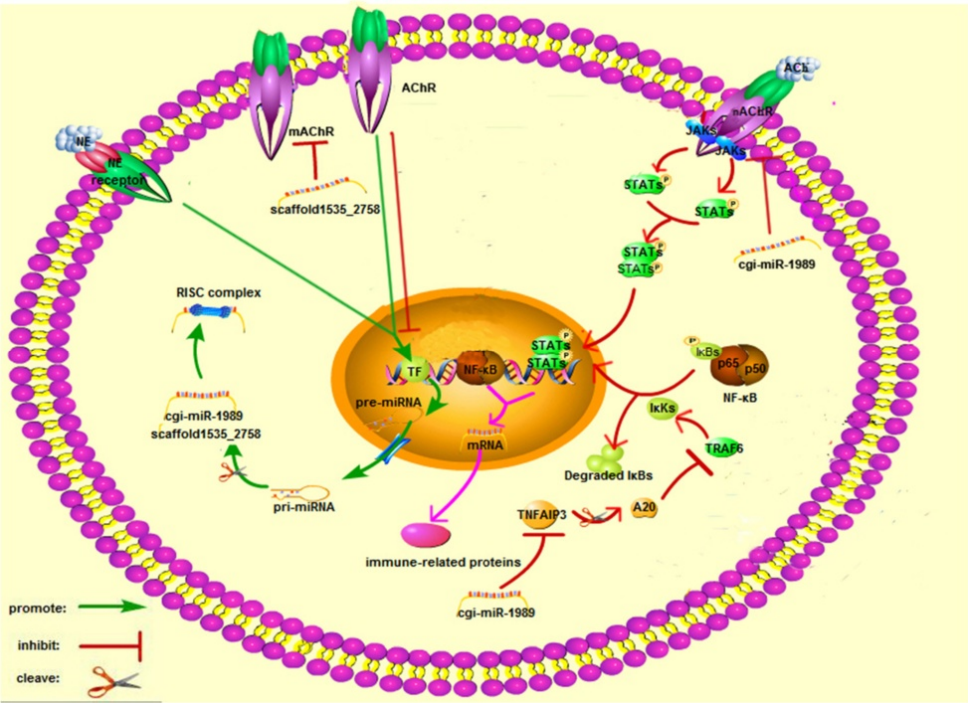
Immunomodulation of ACh and NE were differentiated by ACh and NE responsive NeurimmiRs. NeurimmiR cgi-miR-1989 was found decreased after ACh and increased after NE stimulation and predicted to target oyster transcripts for nAChR and TNFAIP3. Scaffold1535_2758 was found up-regulated by ACh and NE and could modulate mAChR. Those two miRNAs were proposed to contribute largely to the immunomodulation differences between ACh and NE

Given the pathways and genes, the immunomodulation mechanism of ACh and NE might share certain similarities or was sometimes in an neurotransmitter-specific manner [41–44]. This can also be observed in those ACh- and NE-responsive miRNAs from oyster. For example, five out of 38 neurotransmitter-responsive miRNAs (cgi-miR-125, cgi-miR-8, scaffold1144_2255, scaffold1711_599 and scaffold226_18954) showed similar expression pattern after ACh or NE stimulation, and immune-related GO terms including response to stimulus and gene expression were annotated from their target genes (Fig. 10a). Those miRNAs confirmed the perspective that ACh and NE could partly share conserved immunomodulation pathway. On the other hand, 21 miRNAs exhibited different expression pattern after ACh or NE stimulation, with six of them (cgi-miR-182, cgi-miR-242, scaffold1600_212, cgi-miR-1989, scaffold1785_16815 and scaffold625_3183) in an opposite trend. Among them, cgi-miR-1989 was of the most interest as it decreased in ACh group while increased in NE group and was a putative regulator of TNFAIP3 and nAChR (Fig. 11). It was proved that TNFAIP3, a key factor in NF-κB pathway, could attenuate NF-κB activation during the infection [45] while nAChR, one of important ACh receptor, could also modulate the inflammatory responses through both NF-κB and JAK/STAT pathway [46, 47]. Those differentially expressed NeurimmiRs were supposed to result in the differences between immunomodulation of ACh and NE. Moreover, the modulation differences could also be augmented by scaffold1535_2758, a putative regulator of mAChR, which was significantly up-regulated after ACh stimulation and ascended modestly after NE stimulation. It was proposed that scaffold1535_2758 could promote the nAChR-initialed signaling after ACh stimulation while suppress the cholinergic signaling remarkably after NE stimulation by cooperating with cgi-miR-1989. Collectively, those NeurimmiRs synergistically render the host with a closely interacted NEI system in oyster.

Phagocytosis of immunocytes is a highly integrated immune process, accomplished by multiple genes involved in pathogen recognition, signal transducing and immune effector expression [25, 48, 49]. In the present study, the phagocytic rate of oyster haemocytes against *V. splendidus* decreased markedly after ACh and NE stimulation (Fig. 9a), which was consistent with speculations proposed above. Comparatively, the apoptosis rate remained comparable in ACh and NE group (Fig. 9b), possibly owing to the modulatory conflicts in integrin pathway and NF-κB pathway, which were considered as two major players in maintaining cell survival [50, 51]. Notwithstanding, late-apoptosis and necrosis rate decreased significantly after the neurotransmitter stimulation (Fig. 9c), indicating a tendency for ACh and NE in maintaining cell survival in oysters.

## Conclusion

In conclusion, a total of 38 neurotransmitter-responsive miRNAs were identified in oyster with possible immunomodulation roles. A comprehensive NeurimmiR-mediated network targeting PRRs, intracellular receptors, signaling transducers and immune effectors was proposed for oyster haemocytes to maintain the homeostasis of NEI system. Those findings for the first time depicted the interaction between invertebrate NeurimmiRs and the ancient NEI system, which would shed light on the understanding of the stress adaptation mechanisms of oyster.

## Materials and methods

### Oyster culture

Oysters *C. gigas* (averaging 150 mm in shell length, 70 mm in width) were collected from a local farm in Qingdao, China. A narrowed notch was sawed for injection in the closed side of oyster shell, which was adjacent to the adductor muscle [52]. Oysters were maintained in aerated sea water (about 20 °C) for two weeks before use. No specific permits are required for the present study and all experiments were conducted with approval from Experimental Animal Ethics Committee, Institute of Oceanology, Chinese Academy of Sciences, China.

### Neurotransmitter stimulation

A total of 30 oysters were employed for neurotransmitter stimulation experiment and randomly divided into three groups, including PBS control group, ACh stimulation group and NE stimulation group. Oysters in PBS control group received an injection of 50 μL phosphate buffered saline (PBS, 0.14 mol L^−1^ sodium chloride, 3 mmol L^−1^ potassium chloride, 8 mmol L^−1^ disodium hydrogen phosphate dodecahydrate, 1.5 mmol L^−1^ potassium phosphate monobasic, pH 7.4) at their adductor muscle while oysters in ACh and NE group were injected with 50 μL ACh (Sigma, 10^−7^ M in PBS) and NE (Sigma, 10^−7^ M in PBS), respectively. All oysters were returned to the aerated sea water immediately after the injection and sampled at 6 hour post injection. To reduce individual variations and improve result reliability, haemocytes from five individuals were harvested by centrifugation (800 g, 4 °C for 10 minutes) and pooled into one sample. Two replicates were conducted for subsequent sequencing in each group.

Another 45 oysters were also employed and received the same treatment as described above for phagocytosis and apoptosis assay. At 6 h post the injection, haemocytes from five oysters in each group were collected aseptically from the posterior adductor muscle sinus with acid citrate-dextrose anticoagulant agent (22 g L^−1^ sodium citrate, 8 g L^−1^ citric acid, 24.5 g L^−1^ glucose, pH 7.4) at the ratio of 8:1 [53] and resuspended in L15 medium (Gibco) with additional saline solution to 2 x 10^6^ cells mL^−1^ before subjected to phagocytosis and apoptosis assay. All trials were conducted with three replicates.

### Construction and deep sequencing of oyster small RNA libraries

Total miRNAs in the haemocytes were extracted with PureLink miRNA Isolation Kit (Invitrogen) according to the manufacture's protocol. RNAs were quantified by Nanodrop 2000 (Thermo Scientific), and further analyzed by Agilent 2100 Bioanalyzer (Agilent Technologies) for integrity. miRNA samples (about 3 μg) with A260/280 ratio above 2.0 were used for subsequent library construction. The construction of small RNA libraries and the following deep sequencing by Ion Torrent Proton was conducted according to manufacturer's instruction with the brief steps including (1) hybridization and ligation of the total miRNA purified by PureLink miRNA Isolation Kit, (2) reverse transcription to synthesis the cDNA, (3) purification and size-selection of the cDNA, (4) amplification and size-selection of the cDNA, (5) assess the yield and size distribution of the amplified DNA using Agilent 2100 Bioanalyzer, and (6) sequencing the libraries using Ion Torrent Proton P1 chip.

### Identification of oyster miRNAs

Raw data obtained from Ion Torrent Proton was pre-processed by Fastx-toolkit pipeline to get the summary of sequencing quality, nucleotide distributions and length distributions. Then reads with high sequencing quality and ranging from 18–30 nt in length were collected by Fastx-toolkit and aligned to Rfam database [54] and oyster mRNAs. After abandoning reads mapped to either non-miRNAs in Rfam (such as rRNA, snoRNA etc.) or oyster mRNAs, the remaining was then mapped to oyster genome using bowtie-1.00 software [55] and analyzed by miRDeep2 [56] to identify known and novel miRNAs with mature miRNAs and precursor sequences from oyster and other species listed in miRBase [57] as references.

### Expression analysis of miRNA

The copy numbers of oyster miRNAs were calculated by home-made Perl script. The differentially expressed miRNAs were determined by edgeR software using generalized linear models with FDR (false discovering rate) value less than 0.05 [58].The expression levels of miRNAs in each group were estimated by FPKM method. A dendrogram of differentially expressed miRNAs was clustered by Cluster3.0 using FPKM value and displayed by Treeview software.

### Target prediction and GO analysis

The 3'-UTR sequences of oyster genes were obtained based on the genome annotation information and subjected to miRanda [59] for target prediction of those neurotransmitter-responsive miRNAs. Genes were regarded as putative targets if they satisfied the criteria with free energy less than −25 kcal/mol and score value higher than 160.

All oyster protein sequences were aligned to non-redundant database of NCBI by a local blastp algorithm (E-value < 1 x 10^−5^) and further parsed by Blast2GO software [60] for assigning GO terms. GO term distribution of target genes was then calculated and exhibited by WEGO [61].

### Phagocytosis and apoptosis assay after neurotransmitter stimulation

Phagocytosis rate of oyster haemocytes was determined using method modified from previous report [52]. In brief, *Vibrio splendidus* [62], isolated from lesions of moribund scallop *Patinopecten yessoensis* and cultured at 16 °C overnight, were labeled with FITC (Sigma) and diluted into 10^8^ cells mL^−1^ before use. A volume of 200 μL haemocytes (2 x 10^6^ cells mL^−1^) obtained previously was then incubated with same volume of FITC-labeled bacteria for 60 min at room temperature in darkness. Haemocytes were subsequently recollected and resuspended in L15 medium to detect the phagocytosis rate using flow cytometry (BD biosciences) with 10, 000 events counted.

Apoptosis rate was detected using the Annexin V-FITC detection kit (Beyotime) according to the manufacturer’s instructions. In brief, 195 μL diluted haemocytes were incubated firstly with 5 μL Annexin V-FITC for 10 min to label early-apoptotic cells and then stained with 10 μL propidium iodide for 5 min to mark the late-apoptotic or necrotic cells. After recollection and resuspension, the haemocytes were immediately subjected to flow cytometry for apoptosis rate detection (10,000 events countered).

Data obtained were analyzed by Statistical Package for Social Sciences (SPSS) 19.0, followed by one-way analysis of variance (ANOVA) and given as means ± S.D. Differences were considered significant at *P* < 0.05 and marked with different letters (a, b, c etc.).

### Availability of supporting data

The raw sequencing data have been deposited in the Sequence Read Archive (SRA) under BioProject SRR1562260.

## Additional files

Additional file 1: Table S1. Statistics for the distribution of reads filtered in order. (DOCX 15 kb) Additional file 2: Figure S1. Sequence length distribution of the libraries. X axis displays the reads length; Y axis shows the amount (by million reads). (PDF 177 kb) Additional file 3: Table S2. Statistics for the filtered clean reads. (DOCX 13 kb) Additional file 4: Table S3. miRNAs identified in the present study and previous study. (XLSX 20 kb) Additional file 5: Table S4. miRNAs with dual precursors in genome identified in the present study. (XLSX 15 kb) Additional file 6: Table S5. FPKM values of 331 miRNAs identified in *C.gigas*. (XLSX 34 kb) Additional file 7: Table S6. Differentially expressed miRNAs identified by edgeR software at FDR < 0.05. (XLSX 9 kb) Additional file 8: Table S7. Neurotransmitter-responsive miRNAs and their target genes predicted by miRanda. (XLSX 17 kb)
