# Supplementary material for: The comprehensive immunomodulation of NeurimmiRs in haemocytes of oyster Crassostrea gigas after acetylcholine and norepinephrine stimulation
Source: BMC Genomics. 2015 Nov 14;16:942. doi: 10.1186/s12864-015-2150-8 (PMC4650145; doi:10.1186/s12864-015-2150-8)
Supplement: Additional file 1: Table S1. — Statistics for the distribution of reads filtered in order. (DOCX 15 kb) [file 12864_2015_2150_MOESM1_ESM.docx]

Table S1. Statistics for the distribution of reads filtered in order.

|  | **PBS group** | | **ACh group** | | **NE group** | |
| --- | --- | --- | --- | --- | --- | --- |
|  | Library_01 | Library _02 | Library _03 | Library _04 | Library _05 | Library _06 |
| **Total reads** | 50,043,944 | 57,496,512 | 54,732,790 | 60,626,519 | 67,994,185 | 63,618,460 |
| **Quality filtered** | 28,528,178 | 30,331,845 | 28,076,154 | 37,575,049 | 39,336,240 | 36,512,309 |
| **Length filtered** | 22,574,535 | 25,989,407 | 24,072,117 | 31,850,376 | 33,532,483 | 33,051,670 |
| **Unique reads remained** | 1,930,527 | 2,026,646 | 1,592,123 | 2,106,604 | 2,271,484 | 2,462,200 |
