# Supplementary figures and images for: The comprehensive immunomodulation of NeurimmiRs in haemocytes of oyster Crassostrea gigas after acetylcholine and norepinephrine stimulation
Source: BMC Genomics. 2015 Nov 14;16:942. doi: 10.1186/s12864-015-2150-8 (PMC4650145; doi:10.1186/s12864-015-2150-8)

Sequence Length Distribution

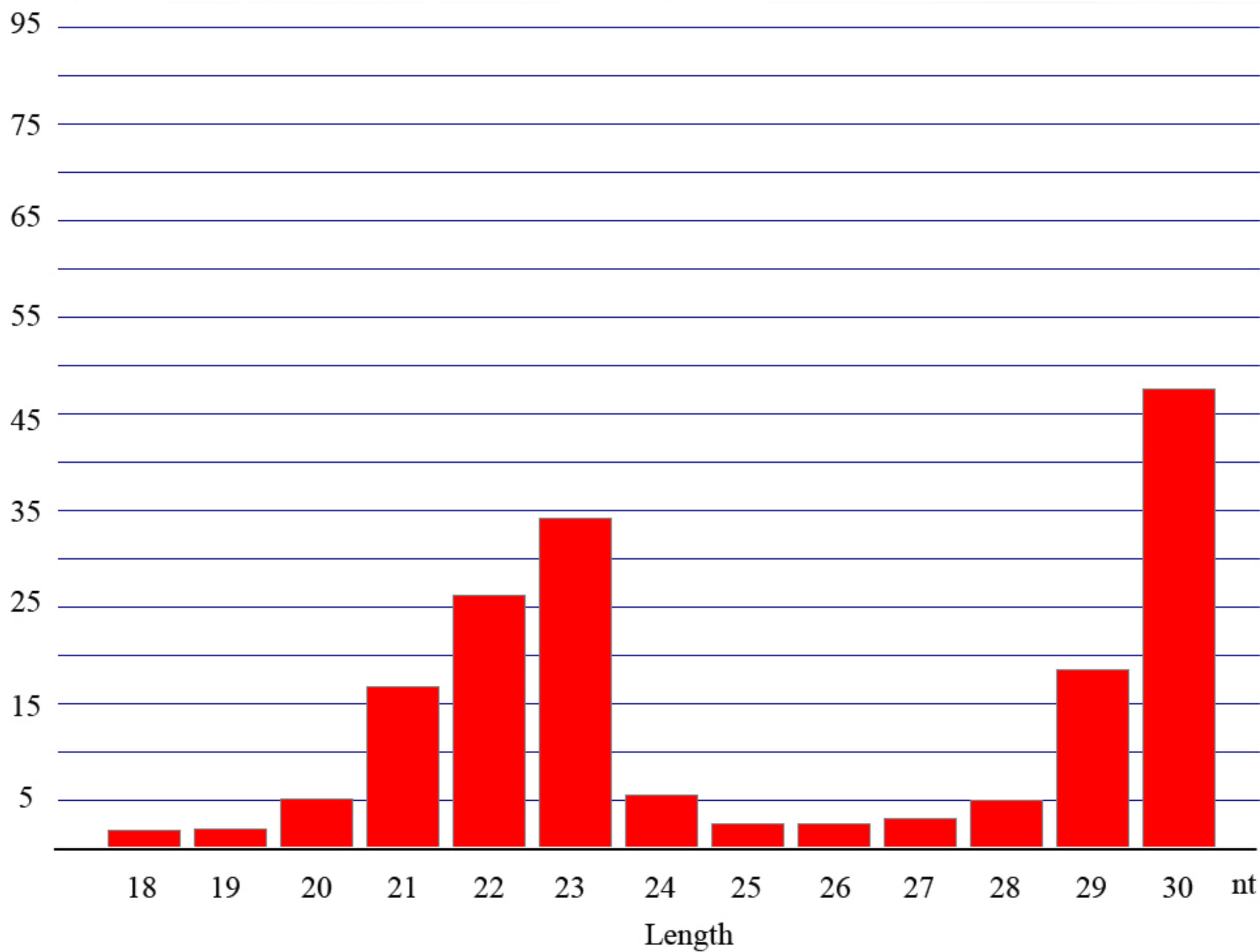

Supplement: Additional file 2: Figure S1. — Sequence length distribution of the libraries. X axis displays the reads length; Y axis shows the amount (by million reads). (PDF 177 kb) [file 12864_2015_2150_MOESM2_ESM.pdf]
