# Supplementary material for: The comprehensive immunomodulation of NeurimmiRs in haemocytes of oyster Crassostrea gigas after acetylcholine and norepinephrine stimulation
Source: BMC Genomics. 2015 Nov 14;16:942. doi: 10.1186/s12864-015-2150-8 (PMC4650145; doi:10.1186/s12864-015-2150-8)
Supplement: Additional file 3: Table S2. — Statistics for the filtered clean reads. (DOCX 13 kb) [file 12864_2015_2150_MOESM3_ESM.docx]

Table S2. Statistics for the filtered clean reads.

| **Total** | **8,556,857** |
| --- | --- |
| **Reads more than 6** | 715,372 |
| **Rfam unmapped** | 684,716 |
| **Oyster mRNA unmapped** | 519,571 |
| **Oyster genome mapped** | 6,820 |
